# Exploring pharmacy provision of medication abortion pills in Nepal: a mixed-methods study of pharmacy workers’ knowledge and practices

**DOI:** 10.1080/26410397.2026.2617716

**Published:** 2026-01-23

**Authors:** Nished Rijal, Angel M. Foster

**Affiliations:** a PhD Candidate, Faculty of Health Sciences, University of Ottawa, Ottawa, Canada; Collaborative for Interdisciplinary Global Abortion Research, Ottawa, Canada; b Professor, Faculty of Health Sciences, University of Ottawa, Ottawa, Canada; Collaborative for Interdisciplinary Global Abortion Research, Ottawa, Canada

**Keywords:** abortion, mifepristone, misoprostol, Nepal, pharmacies

## Abstract

In Nepal, pharmacists are legally permitted to dispense medication abortion drugs with a prescription but provision of mifepristone/misoprostol without a prescription is prohibited. However, one in five Nepalese women seeking medication abortion pills do so directly from pharmacies. This mixed-methods study aimed to understand pharmacy workers’ knowledge of and practices related to medication abortion care and the reasons behind their provision or non-provision of these pills with and without a prescription. We surveyed 489 pharmacy workers and conducted semi-structured interviews with 25 pharmacy workers in major cities in Nepal’s Koshi province. Our survey findings revealed that pharmacy workers are knowledgeable about the legal status of medication abortion (*n* = 414, 85%), medication abortion in general (*n* = 404, 83%), and its timing for use (*n* = 347, 86%). About 16% of pharmacy workers (*n* = 77) reported stocking and selling medication abortion pills, all in the form of combination packages of mifepristone/misoprostol; the average price was Nepali Rupees 660 (USD5). Pharmacy workers who stocked and sold medication abortion pills reported doing so because of the legal permissibility of the practice and community demand; most required a prescription prior to dispensing the medications. Those who did not stock mifepristone/misoprostol cited lack of training, confusion regarding the legal status of medication abortion, business risks associated with provision, and the inconsistent supply of the combination packages. The role of small, community-based pharmacies as service delivery points for medication abortion as a means to expanding access to safe, effective, and accessible care merits further consideration by researchers and policy makers in Nepal.

## Plain language summary

In Nepal, medication abortion drugs are legally permitted to be dispensed by pharmacists if the patient has a prescription. We conducted this study to understand what pharmacy workers in Nepal know about medication abortion and how they dispense pills. We conducted short surveys with nearly 500, and semi-structured interviews with 25, pharmacy workers in Nepal’s Koshi province. Our survey findings revealed that pharmacy workers are knowledgeable about the legal status of medication abortion and when abortion pills can be used. About one in six reported that they carried medication abortion drugs and would dispense them if the patient had a prescription. Those that did not stock medication abortion drugs reported that lack of training, legal and business risks, and supply chain problems influenced their decision. Efforts to work with small, community-based pharmacies to expand access to safe, effective, and accessible medication abortion care in Nepal appear warranted.

## Résumé

Au Népal, la délivrance sur ordonnance de médicaments abortifs par les pharmacies est légale, mais la fourniture de mifépristone/misoprostol sans ordonnance est interdite. Pourtant, une Népalaise sur cinq souhaitant un avortement médicamenteux se procure les comprimés directement en pharmacie. Cette étude à méthode mixte visait à comprendre les connaissances et les pratiques des employés de pharmacie en matière d’avortement médicamenteux et les raisons de leur décision de délivrer ou non ces pilules, avec ou sans ordonnance. Nous avons interrogé 489 employés de pharmacie et mené des entretiens semi-structurés avec 25 d’entre eux dans les principales villes de la province népalaise de Koshi. Les résultats de notre enquête montrent que les employés de pharmacie connaissent le statut légal de l’avortement médicamenteux (*n* = 414, 85%), l’avortement médicamenteux en général (*n* = 404, 83%) et son déroulement (*n* = 347, 86%). Près de 16% des employés de pharmacie (*n* = 77) ont déclaré stocker et vendre des pilules abortives, toujours sous la forme de conditionnement combinant la mifépristone et le misoprostol; le prix moyen était de 660 roupies népalaises (NRS) soit 5 dollars ($ US). Les employés des pharmacies qui stockaient et vendaient des comprimés pour l’avortement médicamenteux ont indiqué le faire en raison de la légalité de cette pratique et de la demande au sein de la communauté; la plupart demandaient une ordonnance avant de délivrer les médicaments. Ceux qui n’avaient pas de mifépristone/misoprostol en stock ont invoqué le manque de formation, le statut légal de l’avortement médicamenteux, les risques commerciaux associés à la distribution et l’approvisionnement irrégulier des conditionnements combinés. Le rôle des petites pharmacies communautaires comme points de service pour l’avortement médicamenteux mérite que les chercheurs et les décideurs népalais s’y intéressent plus en profondeur.

## Resumen

En Nepal, la ley permite que farmaceutas dispensen medicamentos abortivos con receta médica, pero se prohíbe la provisión de mifepristona/misoprostol sin receta. Sin embargo, una de cada cinco mujeres nepalesas que buscan pastillas para inducir el aborto con medicamentos lo hacen directamente por medio de farmacias. El objetivo de este estudio de métodos mixtos era entender los conocimientos y las prácticas de técnicos farmacéuticos con relación a los servicios de aborto con medicamentos y las razones detrás de la provisión o falta de provisión de estas pastillas con o sin receta médica. Encuestamos a 489 técnicos farmacéuticos y realizamos entrevistas semiestructuradas con 25 técnicos farmacéuticos en las principales ciudades de la provincia de Koshi en Nepal. Los hallazgos de nuestra encuesta revelaron que los técnicos farmacéuticos están bien informados sobre el estado legal del aborto con medicamentos (*n* = 414, 85%), el aborto con medicamentos en general (*n* = 404, 83%) y el momento indicado para su uso (*n* = 347, 86%). Aproximadamente el 16% de los técnicos farmacéuticos (*n* = 77) informaron tener en inventario y vender pastillas abortivas, todas en la forma de paquetes combinados de mifepristona y misoprostol; el precio promedio era de 660 rupias nepalíes (NPR) (USD5). Los técnicos farmacéuticos que tenían en inventario y vendían pastillas abortivas informaron que lo hacen debido a que esta práctica es permitida por ley y a la demanda comunitaria; la mayoría exigía una receta médica antes de dispensar los medicamentos. Los que no tenían en inventario mifepristona/misoprostol citaron la falta de capacitación, el estado legal del aborto con medicamentos, los riesgos comerciales asociados con la provisión y el suministro no continuo de los paquetes combinados. La función de las farmacias pequeñas comunitarias como puntos de entrega de servicios de aborto con medicamentos merece consideración adicional por parte de investigadores y formuladores de políticas en Nepal.

## Introduction

Globally, approximately 7.9% of all maternal deaths are the result of unsafe abortion.^1^ Of the nearly 56 million abortions that take place each year, researchers estimate that 45.1% are either “unsafe” or “less safe” and almost all of these abortions take place in countries in the Global South.^2^ Between 1990 and 2019, the global unintended pregnancy rate decreased from 79 to 64 per 1000 women of reproductive age and the proportion of unintended pregnancies ending in abortion increased from 51% to 61%,^3^ indicating that women are exercising more autonomy over their reproductive health outcomes. Comprehensive abortion care is now included in the list of essential health services of the World Health Organization (WHO)^4^ as well as an additional priority in the Minimum Initial Service Package for sexual and reproductive health care in humanitarian settings.^5^

Nepal liberalised its abortion laws in 2002. Women can obtain an abortion in Nepal through 12 weeks of gestation for almost all reasons. Abortion is legally permissible through 18 weeks of gestation if the pregnancy is the result of rape or incest and throughout the entire pregnancy if the woman’s life or physical or mental health is threatened or if the fetus suffers from a serious fetal abnormality or impairment. Approximately 57% of married women of reproductive age use a method of contraception but as many as 21% want to space or limit births but are not using contraception.^6^ Thus, unintended pregnancy is relatively common; a recent estimate placed the national rate at 60 pregnancies per 1000 women aged 15–49 per year.^7^ In 2014, approximately 323,000 abortions took place in Nepal, which translates to 42 abortions per 1000 women aged 15–49.^8^

Medication abortion with mifepristone/misoprostol was introduced in Nepal in 2009 and trained health practitioners in registered health facilities are able to provide abortion services free of charge up to 9 weeks of gestation.* The Department of Drug Administration (DDA), a government regulatory body in Nepal, has approved the mifepristone/misoprostol combination package (one 200 mg tablet of mifepristone and four 200mcg tablets of misoprostol) for medication abortion; these combination packages can be dispensed by providers in publicly funded facilities. The Nepal government has implemented a task-sharing model, authorising trained midwives, auxiliary nurse midwives, nurses, and physicians to provide medication abortion care at government health centres certified for safe abortion. These services are also offered at reduced rates at the clinics operated by non-governmental organisations (NGOs), including Marie Stopes International (MSI) and the Family Planning Association of Nepal (FPAN). Community pharmacies in Nepal can stock and sell medication abortion pills with a prescription but dispensing mifepristone/misoprostol without a prescription is not allowed.

More than two-thirds of women inducing an abortion in Nepal are now do so using medications.^9^ Despite legalisation and government efforts, access to safe abortion services in Nepal remains uneven. As of 2018, medication abortion services had only been expanded to 51 out of 77 districts in the country^10^ and evidence suggests less than half of all abortions (about 42%) occur in government-approved health facilities.^8^ Indeed, the majority of abortions take place outside of the formal health system, are performed by untrained providers and/or in unapproved facilities, or are induced by the pregnant woman herself.^8^ This can result in negative health outcomes; one study found that 68% of women seeking post-abortion care in tertiary-level hospitals had used medications as the primary method of induction and that 89% of these women took unsafe, ineffective, or unknown-quality drugs.^11^

Pharmacies are important actors in the community-based distribution of medication abortion drugs (misoprostol with or without mifepristone). When women experience barriers accessing safe abortion care through the formal health system there is often demand for “extra legal” distribution directly from pharmacies.†^12^ An emerging body of evidence from the Global South, and Asia in particular, suggests that pharmacy provision of abortion medications is common, in both legally permissible and legally restrictive settings.^13–15^ Although the Government of Nepal discourages the sale of medication abortion drugs without a prescription, pharmacies are often the first contact point for those seeking abortion information and services. Indeed, official statistics indicate that pharmacies account for 19% of all abortion services received by women in Nepal,^9^ in part because medication abortion drugs are widely available in pharmacies and drug shops.^18,19^ The DDA, the body that regulates pharmacies in Nepal, estimates that about 50% of the 28,000 pharmacies in Nepal are operating without a licence and are thus technically “illegal”.^20^ Nepal also has about 1100 miles of open borders with India and citizens of both countries can travel freely between the two countries without a visa. India is the third largest producer of medicines globally and there is considerable speculation that unregulated medication abortion drugs – that is, specific brands that have not been approved for sale and distribution – make their way into Nepal from India.^18,21^

A small body of research conducted in Nepal and elsewhere has demonstrated that pharmacy workers often lack essential knowledge on effective medication abortion regimens and rarely offer appropriate counselling.^13,18,22^ However, the literature on Nepalese pharmacy workers’ knowledge of and provision practices related to medication abortion is limited. This mixed-methods study explores the medication abortion knowledge and practices of pharmacy workers in community settings in Nepal and the reasons behind provision and non-provision.

## Methods

**Figure 1.**
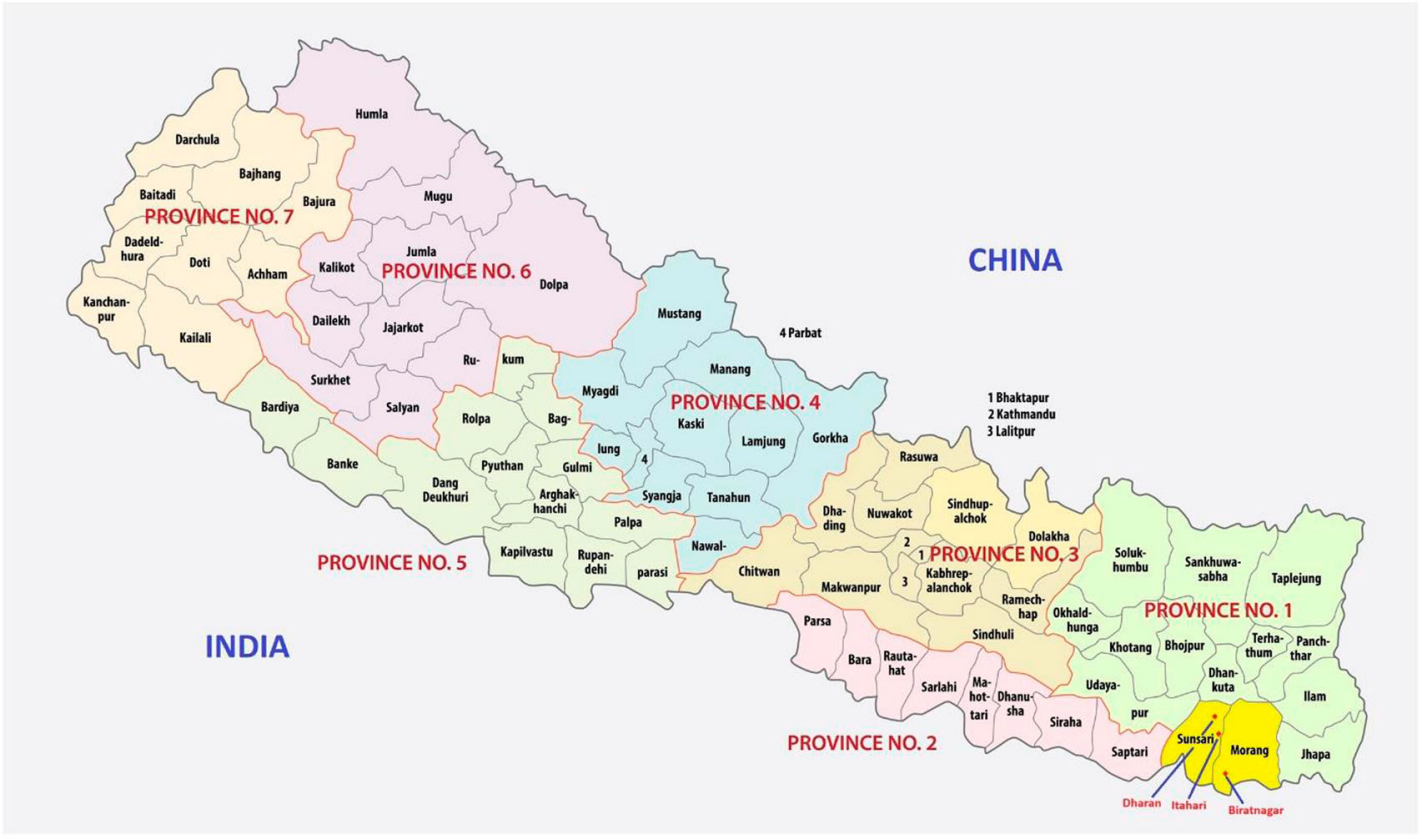
Map of Nepal with study sites in Province No. 1 highlighted

From November 2023 through December 2023 we undertook a concurrent mixed-methods study with two components: a cross-sectional survey of a stratified sample of pharmacies and semi-structured key informant interviews with pharmacy workers. For the purposes of this study, we use “pharmacy worker” to refer to professional groups recognised by the Drug Advisory Committee of the DDA that can sell or distribute prescription drugs^23^ or any pharmacy staff who were dispensing medicines at the pharmacy during our visit. We conducted this study in Morang and Sunsari districts of Koshi Province Nepal due to the limited research on pharmacy-based medication abortion provision in these locations, as well as their high population density, ethnolinguistic diversity, peri-urban setting, and proximity to the Indian border. The proximity to the Indian border exposes these districts to the cross-border supply of unregistered medicines. We covered two sub-metropolitan areas (Dharan and Itahari) and one metropolitan city (Biratnagar) as well as one municipality (Duhabi) and one rural municipality (Budiganga) located between these major cities covering all areas within this 70-kilometre stretch of Koshi Highway. These study areas form three broad clusters: the Dharan cluster covering the Dharan Sub-Metropolitan City, the Itahari cluster covering Itahari Sub-Metropolitan City, and the Duhabi Municipality and the Biratnagar cluster covering Biratnagar Metropolitan City and Budiganga Rural Municipality. We present a map with the study sites marked as Figure 1.

### Cross-sectional survey of pharmacies

The first component of the study involved a cross-sectional survey of pharmacy representatives to document the availability of medication abortion drugs in the study areas. With respect to the sample, DDA maintains an online database of all registered allopathic pharmacies which indicated that there were 1096 registered pharmacies in Biratnagar (*n* = 567), Dharan (*n* = 303), Itahari (*n* = 148), Duhabi (*n* = 39) and Budiganga (*n* = 39) in late 2023. As only half of pharmacies in Nepal are licensed and registered with the DDA,^20^ we estimated that there were approximately 2200 pharmacies in these five areas. Our eligibility criteria included all retail pharmacies selling allopathic medicines located in the aforementioned cities, irrespective of their DDA registration status. We used a stratified randomised process to select 489 pharmacies (roughly 20–25% of registered and unregistered pharmacies); we based stratification on municipality and the number of pharmacies we selected was proportional to the number of pharmacies registered in each area.

We trained six local survey administrators to collect information using a questionnaire in Nepali through Kobo Toolbox on smartphones. The research team finalised the survey instrument based on feedback from pretesting in 12 pharmacies. From November 24, 2023 to December 1, 2023, the survey administrators operated through a south-to-north and east-to-west grid, contacting roughly every third pharmacy they encountered, a strategy informed by previous studies.^18,24,25^ In cases where pharmacies were closed, eligible respondents were not available, or a pharmacy worker did not consent to the survey, the survey administrators moved to the immediate next pharmacy.

After identifying an appropriate individual in the pharmacy (for example, the owner or pharmacy staff) and obtaining written consent, the survey administrator used a structured questionnaire and asked questions related to the following domains of inquiry: pharmacy operations (including licensure), client characteristics, availability and cost of medication abortion drugs, provision practices related to medication abortion drugs (including volume), if applicable, and training of personnel. We assigned each included pharmacy a unique identifier. The survey administrators uploaded the completed questionnaires at the end of the day or whenever they had access to the Internet.

### Semi-structured interviews with pharmacy workers

We conducted semi-structured key informant interviews with 25 pharmacy workers, who did not participate in the survey. We purposively selected pharmacy workers from two categories of pharmacies – those whose representatives reported dispensing medication abortion pills and those whose representatives reported not dispensing the medications – to understand the reasons behind provision or non-provision. NR, a PhD student in Population Health at the University of Ottawa and native of Nepal, conducted the key informant interviews in Nepali after receiving training from his supervisor, AMF, an American medical doctor and medical anthropologist with extensive experience leading qualitative studies related to medication abortion.

We developed a semi-structured interview guide specifically for this study to understand participants’ backgrounds and the characteristics of their pharmacies, their training and experiences providing medication abortion, their reflections on the factors affecting the availability or non-availability of medication abortion services, and their perspectives on ways that pharmacy-based services could be improved. Between November and December 2023, we approached pharmacy workers in different types of positions in different locations in our study area. After explaining the study's purpose and obtaining informed written consent, we audio-recorded all but one interview. One key informant consented to the interview but not to the audio-recording and we took extensive notes instead. NR took notes during each interview and prepared memos immediately afterward. We used thematic saturation as our endpoint;^26,27^ we suspected we had reached thematic saturation after conducting 22 interviews and did three additional interviews as confirmation. On average, interviews lasted about 22 minutes.

### Data analysis

Initially, we analysed each component of the study separately. We conducted a preliminary audit of the survey data in the Kobo Toolbox; we then exported the collected data from the Kobo Toolbox into XLS format. After further cleaning the data, we conducted our analysis using IBM Statistics SPSS 29.0. We used descriptive statistics to analyse pharmacy demographics, medication abortion availability and cost, and the knowledge and practices of pharmacy workers.

We analysed the semi-structured interviews for content and themes^28,29^ using an iterative process. NR translated the audio-recordings, memos, and notes into English and then used ATLAS.ti 24 to manage the data. We developed an initial codebook in light of the relevant literature, research questions, and study instruments and added emergent codes as we familiarised ourselves with the data, thus using both inductive and deductive techniques.^28,29^ Regular discussions between NR and AMF guided our interpretation of the study findings. The final step in the analytic process centred on identifying concordant and discordant findings between the different study components. This step served as a form of triangulation.^30^

### Ethical considerations

We received ethics approval for this study from the Research Ethics Board at the University of Ottawa (S-04-19-3909, dated June 7, 2023). Additionally, we received ethics approval from the Nepal Health Research Council (417/2023, dated October 5, 2023). We have masked all personal identifiers from the individual pharmacy workers and their associated pharmacies throughout the manuscript. We have organised our results around the domains of inquiry.

## Results

### Socio-demographic characteristics of the respondents and their pharmacies

We completed surveys with 489 pharmacy workers distributed in three clusters in Nepal’s Koshi province: Biratnagar (*n* = 184), Dharan (*n* = 163), and Itahari (*n* = 142). The mean age of our participants was 33.8, with minimum and maximum ages of 18 and 67. About three-quarters (*n* = 370, 76%) of the respondents identified as men and the rest (*n* = 119, 24%) as women. In terms of highest educational qualification, the largest share of respondents (*n* = 196, 40%) had a Diploma in Pharmacy followed by a Community Medicine Assistant certification (*n* = 83, 17%), bachelor’s degree (*n* = 62, 13%), and Health Assistant certification (*n* = *n* = 57, 12%).

Participants from almost all of the pharmacies reported having less than 100 client visits per week for any/all services (*n* = 474, 97%) and as having been registered (*n* = 483, 99%) under the DDA. A little more than half (*n* = 274, 56%) of the respondents were the owners of the pharmacy and the rest (*n* = 215, 44%) were staff working in the pharmacy. About half (*n* = 244, 50%) of the respondents had 1–5 years of experience working at the pharmacy. We present the characteristics of the respondents and their affiliated pharmacies in Table 1.

**Table 1.** Characteristics of the cross-sectional survey respondents and their pharmacies in Nepal’s Koshi province (*N* = 489)

Demographic characteristics of pharmacy worker participants (N = 489)	*N*	%
***Gender***		
Man	370	75.7
Woman	119	24.3
***Average age (in years)***	33.8 ± 10.5	
***Age (in years)***		
18–30	239	48.9
31–45	176	36
>45	74	15.1
***Highest educational qualification***		
Diploma in Pharmacy	196	40.0
Community Medicine Assistant	83	17.0
Bachelor’s Degree	62	12.7
Health Assistant	57	11.7
High School	21	4.3
Secondary Education Examination or below	21	4.3
Master’s Degree	23	4.7
Auxiliary Nursing Midwife	14	2.9
Lab assistant/technician	9	1.8
MBBS	2	0.4
Staff Nurse	1	0.2
***Owner of the pharmacy***		
Yes	274	56.0
No	215	44.0
***Duration of work at the pharmacy***		
Less than 1 year	63	12.9
1 year-5 years	244	49.9
More than 5 years	182	37.2
**Pharmacy characteristics (*N*** **=** **489)**		
***Average clients visit per week for any services***		
1–100	474	96.9
101–200	14	2.9
201 or more	1	0.2
*** Pharmacy registration under the Department of Drugs Administration***		
Yes	483	98.8
No	5	1.0
Don’t know	1	0.2
** *Location of the pharmacy***		
Biratnagar	184	37.7
Dharan	163	33.3
Itahari	142	29.0

Of the 25 key informants, 10 were from Dharan, nine were from Itahari, and six were from the Biratnagar clusters. Twenty-two participants identified as men and the other three as women. In terms of qualifications, nine held a Community Medicine Assistant (CMA) certificate, nine had a Diploma in Pharmacy combined with a CMA or Health Assistant degree, two were lab technicians, two were Health Assistants, one was an Auxiliary Nursing Midwife (ANM), and one had education below the Secondary School Examination level.

### Pharmacy workers’ knowledge of medication abortion

As illustrated in Table 2, the majority (*n* = 414, 85%) of our survey respondents accurately reported that abortion is legal in Nepal and 83% (*n* = 404) reported general knowledge and familiarity with medication abortion. About half of the respondents (*n* = 192, 48%) reported that medication abortion can be performed up to and including nine weeks of pregnancy and 38% (*n* = 155) reported medication abortion is allowed for less than nine weeks of pregnancy. The most commonly reported possible complications from medication abortion were blood loss requiring transfusion/stabilisation (*n* = 335, 69%) followed by continued pregnancy (*n* = 127, 26%), incomplete abortion (*n* = 99, 20%), and retained products of conception (*n* = 57, 12%). A large majority (*n* = 465, *n* = 95%) of the respondents had not received any formal training or orientation on medication abortion. Among the few who had (*n* = 20, 4%), respondents reported receiving orientation from various organisations, including the Nepal Contraceptive Retail Sales Company, Department of Drug Administration, Population Services International, FPAN, MSI, and government health centres. One respondent mentioned receiving medication abortion training in India. About two-thirds (*n* = 324, 66%) of the respondents could calculate correctly the duration of pregnancy based on the first day of the last menstrual period.

**Table 2.** Pharmacy workers’ knowledge of medication abortion in Nepal’s Koshi province (*N* = 489)

Knowledge assessment areas	*N*	%
***Abortion legality in Nepal (N*** ***=*** ***489)***		
Yes, legal in the first trimester	414	84.7
No, abortion is not legal	46	9.4
Don’t know	29	5.9
***General familiarity with medication abortion (N*** ***=*** ***489)***		
Yes	404	82.6
No	59	12.1
Don’t know	26	5.3
***Medication abortion is legally permitted through (N*** ***=*** ***404^a^)***		
Less than nine weeks (any duration under 63 days)	155	38.4
Up to and including nine weeks (63 days)	192	47.5
More than nine weeks (any duration over 63 days)	31	7.7
Don’t know	26	6.4
***Possible complications of medication abortion (N*** ***=*** ***489^b^)***		
Blood loss requiring a transfusion/stabilization	335	68.5
Incomplete abortion	99	20.2
Ongoing pregnancy	127	26.0
Retained products of conception	57	11.7
Other	38	7.8
Don’t know	87	17.8
***Ever received training/orientation on medication abortion (N*** ***=*** ***489)***		
Yes	20	4.1
No	465	95.1
Don’t know	4	0.8
***Calculation of duration of pregnancy (N*** ***=*** ***489)***		
Correct response (by first day of the last menstrual period)	324	66.3
Incorrect response	110	22.5
Don’t know	55	11.2

^a^Only asked to those who knew about medication abortion by pills

^b^Percentages may not add to 100% due to multiple-choice answers

Most of our semi-structured interview participants were knowledgeable and appreciative of the fact that existing laws in Nepal allowed them to store and sell medication abortion pills. However, they had mixed knowledge levels on the duration of pregnancy up to which medication abortion can be offered legally. As stated by a male pharmacy worker from Dharan,

*“Nepal government legislation allows abortion. It is not illegal. It mentions that medication abortion can be provided within certain weeks and that’s why medicines are available at the pharmacies. That’s it. The government has provided legislation. Abortion is allowed for up to 12 weeks. You can either do the [manual vacuum aspiration] or use the [medication abortion] kit”*.

Consistent with our survey participants, most of our interviewees had not received any training or orientation in medication abortion and explained that their pharmacy did not have trained or qualified personnel to dispense these medicines. They expressed a lack of confidence in their ability to dispense medication abortion drugs in the correct dosage and an interest in receiving this type of training. Even those who has received limited training described it as insufficient. However, a number of our participants believed this was a missed opportunity. As explained by a male interviewee working in a pharmacy in Itahari, “*I haven’t received any training on this [medication abortion] which is very unfortunate from the side of the Nepal government. Many clients are coming to seek medication abortion services daily however there are no training or facilities for these services from the Nepal government”*.

### Pharmacy workers’ practices related to medication abortion

About 16% (*n* = 77) of our survey respondents reported stocking and dispensing medication abortion pills from their pharmacies. All of these respondents provided the mifepristone/misoprostol combination pack. Those who reported providing care offered medication abortion pills to an average of 1.58 clients per week. Of those who reported stocking medication abortion drugs, the average on hand supply was 3.55 mifepristone/misoprostol combination packages and the average price was Nepali Rupees (NRS)660 (about USD5).

Table 3 illustrates the providing practices of our respondents. Of those dispensing directly to abortion seekers, most (*n* = 66, 86%) reported offering the correct mifepristone/misoprostol regimen (one 200 mg tablet of mifepristone used orally on day one, followed by four tablets (800 mcg) of misoprostol 24–48 h later used vaginally, buccally, or sublingually). About two-thirds (*n* = 47, 61%) of these respondents reported routinely providing clients with information on when/how to take the drugs. Other advice included information on possible side effects (*n* = 19, 25%), possible complications (*n* = 9, 12%), follow-up (32, 42%), referral in case of complications (*n* = 8, 10%), and additional medications (*n* = 13, 17%). Advice on post-abortion contraception was almost non-existent (*n* = 1, 1%).

**Table 3.** Medication abortion practices of pharmacy workers who stock and dispense medication abortion drugs in Nepal’s Koshi province (*N* = 77)

Practices of pharmacy workers	*n*	%
***Types of pills offered for medication abortion (N*** ***=*** ***77)***		
Mifepristone/misoprostol combination package	77	100
Misoprostol alone	0	0
Other	0	0
***Recommended regimen (N*** ***=*** ***77)***		
Correct response	66	86
Incorrect response	9	13
Don’t know	2	3
***Information required from clients seeking medication abortion (N*** ***=*** ***77^a^)***		
Doctor’s prescription	72	94
Client’s age	14	18
Confirmation of pregnancy	21	27
Gestational age	12	16
Husband/partner consent	15	20
***Information offered to clients who purchase medication abortion pills (N*** ***=*** ***77^a^)***		
Drug regimen	40	52
Side effects	19	23
Complications	9	12
Referral in case of complications	8	10
Follow-up	32	42
Post-abortion contraception	1	1
Interaction with other medications	13	17
Other	3	4
***Keeps medical record for those obtaining medication abortion pills (N*** ***=*** ***77)***		
Yes	6	8
No	70	91
Don’t know	1	1

^a^Percentages may not add to 100% due to multiple-choice answers

Almost all pharmacy workers reported that medication abortion clients were required to have a prescription (*n* = 72, 94%). Some pharmacy workers also required clients to offer proof of pregnancy (*n* = 21, 27%), document a husband/partner’s consent (*n* = 15, 20%), provide their age (*n* = 14, 18%), and/or establish the gestational age of the pregnancy (*n* = 12, 16%). Only a small number (*n* = 6, 8%) reported having some form of record-keeping system for dispensing medication abortion drugs, such as tracking the number of pills stocked or dispensed, date of dispensation, or client information.

Of our interviewees, 14 of 25 reported stocking and dispensing medication abortion pills. The pharmacy workers emphasised that dispensing medication abortion drugs was legally permitted and by dispensing the medications they were meeting a pressing community demand. Several interviewees also highlighted situations in which medication abortion care was particularly important, such when a young unmarried girl presented with an unintended pregnancy, the pregnancy resulted from an extra-marital affair, or contraceptive failure had occurred. A male pharmacy worker from Biratnagar put it succinctly, “*When there is an unintended pregnancy, there is a need for abortion services*”. Pharmacy workers reported offering medication abortion care to both known clients from the community and abortion seekers who travelled from other areas, likely to ensure that the pharmacy worker was outside of their social and familial circles.

The pharmacy workers that we interviewed who dispensed medication abortion drugs all reported that they only do so if the client has a prescription. They also all noted that they only dispense combination packages that have been registered and approved by the government of Nepal, including Synopill®, Mariprist® or Pregno Mistol®. Participants indicated that the price ranged from NRS500-700 depending on the manufacturing company; a few reported that they collect a standard profit margin of 16% on all medicines including medication abortion pills. However, one female pharmacy worker reported that she was aware of at least one pharmacy that was price gouging, charging NRS3000 (USD22) for a single combination package. Several interviewees speculated that the variation in price was related to the “desperation” of the client. As one male pharmacy worker from Dharan stated,

*“If we sell via prescription, we will just have the normal percentage (of profit). If it is an emergency and if sold secretly without a prescription, you can sell in whatever amount you like, right? When people are in an emergency, they are willing to pay any amount”*.

Eleven of our 25 key informants did not stock or dispense medication abortion pills. These participants cited lack of training, their belief that Nepali law does not allow for pharmacy dispensing, potential risks to the business, and inconsistent supplies of the medications as reasons for not offering this service. As a male pharmacy worker from Itahari explained, “*Until we have the authority and training from the Nepal government, we are unable to provide [medication abortion] services. We need mifepristone which I don’t have”*.

But all participants who were not currently stocking and dispensing medication abortion drugs were willing to do so, if they were confident the law permitted the practice. They also stressed the need to build the capacity to provide the services. They signalled eagerness to expand abortion services in their pharmacies as expressed by a male pharmacy worker from Itahari:

*“If our staff had relevant training, and if we could bear the risk, we could [provide] these services. One helpless thing for us is the [medication abortion] clients come to us, but we cannot provide them with the service. If health professionals at the pharmacy like Health Assistants and [Auxiliary Health Workers] … well I don’t know the guidelines … if they can be provided with medication abortion training, they have the skills to manage simple cases in case of emergency, [then medication abortion] would be more accessible for all, right? If we can provide this service through this channel, unsafe abortion could be prevented. The client would be able to get these services easily. If the concerned authority could manage the training in this regard, this service could be expanded in a better way.”*

But even if they did not provide medication abortion services, our pharmacy worker interviewees stated that they would not let clients leave empty-handed. Instead, they offered clients information on where they could access safe abortion care, including at MSI clinics, FPAN clinics, government health centres, or private hospitals, depending on proximity. Some of our interviewees reported that NGOs like MSI and FPAN had asked them to refer clients to their clinics and offered financial compensation for referrals. As explained by a male pharmacy workers in Itahari, “*Marie Stopes staff came to us and informed us about the services they offer. So, [my] staff refer them there and they [Marie Stopes] also provide us with a commission. Thus, there is also motivation plus it’s safe as well, right? There is no risk as well. So, we send them there.”*

## Discussion

Our findings shed light on the availability of mifepristone/misoprostol and pharmacy workers’ medication abortion-related knowledge and practices in the Koshi province of Nepal. Previous pharmacy-based studies in Nepal suggest few pharmacy workers knew of mifepristone (4%) and misoprostol (5%) in 2005, prior to government approval of medication abortion. This increased to 30% and 27% respectively in 2009, the year medication abortion was approved, and further increased to about 50% for both medications in 2018, almost a decade after the approval of mifepristone/misoprostol.^22^ Our findings suggest more than three-quarters (83%) of pharmacy workers in Koshi province now know about medication abortion by pills, demonstrating the continuation of this trend. A study conducted in 2005, four years before the approval of mifepristone/misoprostol in Nepal, identified 52 different types of allopathic medicines available for abortion purposes.^19^ Our findings indicate that the medication abortion drugs stocked and dispensed by pharmacy workers universally consisted of the mifepristone-misoprostol combination packages reflecting changes in the availability of abortifacients.

Although only 16% of the pharmacy workers in our sample reported dispensing medication abortion pills to abortion seekers (almost always with a prescription), the pharmacy workers that we interviewed all indicated that they referred clients to abortion providing facilities. Given that one in five Nepalese women seeking medication abortion pills first approach a pharmacy,^9^ this finding is encouraging. This practice also has the potential to reduce unsafe abortion; consistent with the global evidence,^31–33^ research from Nepal indicates that when women are denied legal abortion services they often seek services “elsewhere”.^34^

But our findings suggest that pharmacy workers in the Koshi province in Nepal are keen to stock and dispense medication abortion pills. Existing literature suggests pharmacies can offer abortion care that is private, proximate, and affordable, especially when compared to clinics and formal health facilities in rural areas.^11,13,22^ The pharmacy workers in our study who are dispensing medication abortion pills all reported that they were responding to community need and, even in the absence of formal training, were generally providing correct information, although the price of pills varied. Given uneven distribution of government-funded medication abortion services,^35^ community-based pharmacies remain an important option for expanding access to safe care. However, without formal training and education on medication abortion, pharmacy workers reported lacking the confidence to offer the service.

The recent federalisation of Nepal, which aims to devolve authority to provinces and municipalities as a way of improving service delivery and empowering historically marginalised communities, could open avenues for training and orienting pharmacy workers on the provision of medication abortion care. Our study found pharmacy workers have diverse qualifications, including Diplomas in Pharmacy, CMAs, Health Assistants, and ANMs. Evidence from Nepal suggests that mid-level providers and trained pharmacy workers can safely deliver accurate medication abortion information and services.^36–38^ The federal government could expand its current criteria to include all pharmacy workers identified by the Drugs Advisory Committee^23^ and other mid-level providers as eligible to receive medication abortion training. Standardised, mandatory training programmes should be created for pharmacy workers who intend to offer medication abortion services. The Right to Safe Motherhood and Reproductive Health Act 2018,^39^ which currently restricts safe abortion services to certified providers in designated health facilities, could be revised to extend the provision of medication abortion care beyond clinic settings to include other providers. The provincial government could also lead the training for pharmacy workers, while the local government could provide ongoing monitoring and supportive supervision of pharmacies within the safe abortion network to ensure service quality and adherence to national standards.

The WHO’s 2022 abortion care guidelines state that abortion service delivery in the first trimester with minimal medical supervision is safe and effective and can significantly increase access.^38^ The same guidelines state that pharmacy workers have the required competencies to dispense medication abortion drugs for use through 10 weeks and note that an ultrasound is not a requirement for early medication abortion care.^40^ The promise of mifepristone/misoprostol to expand access to safe abortion care has been limited in many countries by non-evidence-based regulations, such as requiring a pre-abortion ultrasound or limiting dispensing to physicians, and burdensome health system requirements, even in countries with liberal abortion laws.^41–43^ Although local governments have the authority to approve the certification of safe abortion sites, they are not able to expand the definition of a safe abortion site.^44^ The federal government could consider expanding this definition to include pharmacies with workers that have been trained and have the capacity to dispense. Simultaneously, local governments could prioritise capacity-building programmes for all pharmacies under their jurisdiction. These programmes could focus on critical aspects of medication abortion care, such as evidence-based dispensing practices and assisting pharmacies in identifying suitable referral pathways for managing complications. This may be an especially important strategy in areas of the country with few clinical services.

### Limitations

The quantitative component of this paper is descriptive in nature and thus we did not develop or test a hypothesis. Although we estimated that our sample represented about 20% of the registered and unregistered pharmacies in the study areas, the overwhelming majority of our survey respondents reported that the pharmacy was registered with the DDA. This means we either under-sampled non-registered pharmacies or participants incorrectly reported the status of their pharmacy, or both. While the DDA maintains a database of retail pharmacies, the addresses in the database were not up to date and thus we are not able to consistently verify the registration status of pharmacies in our sample. The quantitative component of the research may be generalisable to other urban and peri-urban areas of Nepal, but may not be reflective of situations in rural regions of Nepal. The qualitative component of this research is not intended to be generalisable, as is the case with all qualitative research. Despite these limitations, this study offers new evidence and thus can inform directions for future studies.

## Conclusion

Our findings suggest pharmacy workers in Nepal are willing to provide safe abortion information and services and a minority already are stocking and dispensing medication abortion pills. Medication abortion capacity-building initiatives with pharmacy workers have the potential to reduce the number of women who turn to unsafe abortion methods and increase the accessibility of medication abortion pills. The recent federalisation of Nepal that supports initiatives that are locally responsive could open avenues for training pharmacy workers on the provision of medication abortion care and the delegation of authority to local governments to designate safe abortion sites could result in more points of service delivery and expanded access.

## Author contributions

*NR and AMF conceptualised and designed the study protocol and data collection instruments. NR conducted the fieldwork and analysed data with guidance from AMF. NR wrote the first draft and AMF revised the manuscript. Both authors reviewed and approved the final version for journal submission.*

## Data Availability

All relevant data are within the paper. Study instruments and anonymised data are available from the corresponding author, AMF, upon reasonable request.

## Footnotes

* Consistent with the evolving terminology surrounding abortion in English,^16,^^17^ we use the term “medication abortion” throughout this manuscript. Other guidelines and professional societies often refer to this type of abortion as “medical abortion.”.

† We use the term “extra legal” to describe practices that may not be explicitly legally permitted or prohibited or may involve conflicting laws, statutes, and regulations thereby introducing legal complexity.

## Provenance statement

The article was not commissioned and went through external peer review.
